# Spatial Structure of Seagrass Suggests That Size-Dependent Plant Traits Have a Strong Influence on the Distribution and Maintenance of Tropical Multispecies Meadows

**DOI:** 10.1371/journal.pone.0086782

**Published:** 2014-01-31

**Authors:** Jillian L. S. Ooi, Kimberly P. Van Niel, Gary A. Kendrick, Karen W. Holmes

**Affiliations:** 1 School of Plant Biology, Faculty of Natural and Agricultural Sciences, The University of Western Australia, Perth, Western Australia, Australia; 2 School of Earth and Environment, Faculty of Natural and Agricultural Sciences, The University of Western Australia, Perth, Western Australia, Australia; 3 The UWA Oceans Institute, The University of Western Australia, Perth, Western Australia, Australia; 4 Department of Geography, Faculty of Arts and Social Sciences, Universiti Malaya, Kuala Lumpur, Malaysia; 5 Centre for Ecohydrology, School of Environmental Engineering, The University of Western Australia, Perth, Western Australia, Australia; Dauphin Island Sea Lab; University of South Alabama, United States of America

## Abstract

**Background:**

Seagrass species in the tropics occur in multispecies meadows. How these meadows are maintained through species co-existence and what their ecological drivers may be has been an overarching question in seagrass biogeography. In this study, we quantify the spatial structure of four co-existing species and infer potential ecological processes from these structures.

**Methods and Results:**

Species presence/absence data were collected using underwater towed and dropped video cameras in Pulau Tinggi, Malaysia. The geostatistical method, utilizing semivariograms, was used to describe the spatial structure of *Halophila* spp, *Halodule uninervis, Syringodium isoetifolium* and *Cymodocea serrulata*. Species had spatial patterns that were oriented in the along-shore and across-shore directions, nested with larger species in meadow interiors, and consisted of multiple structures that indicate the influence of 2–3 underlying processes. The Linear Model of Coregionalization (LMC) was used to estimate the amount of variance contributing to the presence of a species at specific spatial scales. These distances were <2.5 m (micro-scale), 2.5–50 m (fine-scale) and >50 m (broad-scale) in the along-shore; and <2.5 m (micro-scale), 2.5–140 m (fine-scale) and >140 m (broad-scale) in the across-shore. The LMC suggests that smaller species (*Halophila* spp and *H. uninervis*) were most influenced by broad-scale processes such as hydrodynamics and water depth whereas large, localised species (*S. isoetifolium* and *C. serrulata*) were more influenced by finer-scale processes such as sediment burial, seagrass colonization and growth, and physical disturbance.

**Conclusion:**

In this study, we provide evidence that spatial structure is distinct even when species occur in well-mixed multispecies meadows, and we suggest that size-dependent plant traits have a strong influence on the distribution and maintenance of tropical marine plant communities. This study offers a contrast from previous spatial models of seagrasses which have largely focused on monospecific temperate meadows.

## Introduction

In tropical marine ecosystems, seagrass species occur in dense, well-mixed stands where habitat partitioning is subtle and less defined than in temperate waters. How these multispecies meadows are maintained through species co-existence and what their ecological drivers may be has been an overarching question in seagrass biogeography (see [1]). The three broad categories of ecological drivers in plant communities – life strategy and morphology, interspecific interactions within a community, and response to environmental gradients [2] – operate together to produce an overall combined effect, resulting in the non-random development of species gradients and patches across the habitat range (i.e. *spatial structure*). Thus, descriptions of spatial structure provide insight into the relationships between biological patterns and processes [3] and is a useful starting point for understanding what drives species distributions.

Seagrass species distributions have been linked to differential rates and strategies of growth, reproduction and dispersal [4], [5], [6], [7], interspecific competition [8], [9] and environmental gradients such as water depth, light, temperature and hydrodynamics [10], [11], [12], [13], [14]. Following hierarchy theory, Legendre [15] suggested that physical processes create spatial structure in biological systems at broad scales, while contagious biotic processes (e.g. reproduction, biological interactions, food availability) cause further structuring at finer scales. In the case of seagrasses, Vermaat [1] argued for light and hydrodynamics as the prime physical processes that determine meadow extent by governing depth penetration and dispersal (i.e. broad-scale drivers), while implying that clonal growth strategies and physical disturbance by herbivory and storms maintain the characteristic patchiness of meadows (i.e. finer-scale drivers). However, the link between these underlying processes and species patterns remains inconclusive because very few studies have been spatially explicit. Studies have more commonly focused on the seagrass shoot or ramet at distances of centimetres to metres. This creates a problem in scaling-up from local process to broader-scale patterns [13], [16], [17], leading to the observation that spatial scales of field measurement have rarely been aligned with specific ecological processes in seagrass research [18].

Seagrass studies that have explicitly linked processes to spatial structure do so either through cross-scale studies that identify scale-dependent differences in species distribution and abundance, or by using techniques which explicitly incorporate spatial structure, e.g. variogram modelling. Both approaches have demonstrated spatial structures in the distribution of seagrasses across multiple spatial scales, but what we know is drawn mostly from studies in meadows where species differentiation in space is strong and species form individual monospecific meadows. Examples include *Amphibolis griffithii*, *Posidonia coriacea*, and *P. sinuosa* occurrence in southwest Australia [16], [19]; *Zostera tasmanica* morphology and bed architecture in southeast Australia [20]; *Zostera marina* and *Halodule wrightii* occurrence in Beaufort, southeast USA [13]; *H. decipiens* occurrence in Florida, southeast USA [21]; *P. oceanica* plant and meadow architecture in the Mediterranean [22], [23], [24], [25]; the within-meadow genotypic distribution of *P. oceanica* [26], and the kinship structure of *Zostera marina* [27].

In contrast, spatially-explicit patterns and processes in multispecies meadows have received less attention. Our study addresses this gap by using geostatistics to model the spatial structure of four co-existing tropical species in a forereef system in Pulau Tinggi, Malaysia. Geostatistical techniques were selected because they have the potential to capture small differences in spatial structure, and in doing so provide insight into the spatial relations of species in their environment [28]. We ask two questions (1) what is the spatial structure of *Halophila* spp., *Halodule uninervis, Syringodium isoetifolium* and *Cymodocea serrulata* distribution in Pulau Tinggi?; and (2) what are the critical spatial scales at which the spatial structures of taxa are most evident? Based on these spatial scales, we make suggestions about what some of the potential ecosystem drivers may be for this study area.

## Methods

### Ethics Statement

This project was conducted under the Economic Planning Unit of Malaysia Research Permit no. UPE: 40/200/19/2351 and the Marine Parks Department of Malaysia Research Permit no. JTLM (S) 620-2/1/1 Jld 2(2).

### Study Area

The field survey was conducted from 15 April to 15 May 2009 in Pulau Tinggi (pulau = island), a continental island located 12 km off the southeast coast of Peninsular Malaysia. It is ringed by fringing coral reefs, with subtidal seagrass meadows in the forereef zone, i.e. on the seaward side of the coral reefs, extending down to a depth of approximately 10 m. The multispecies meadows consisted of *Halophila ovalis* (R. Br.) Hooker f., *Halodule uninervis* (Forsskal) Ascherson, *Cymodocea serrulata* (R. Brown) Ascherson, *Syringodium isoetifolium* (Ascherson) Dandy, *Halophila minor* (Zollinger) den Hartog, *Halophila decipiens* Ostenfeld, and *Halophila spinulosa* (R. Brown) Ascherson [29].

### Data Collection

**Figure 1:**
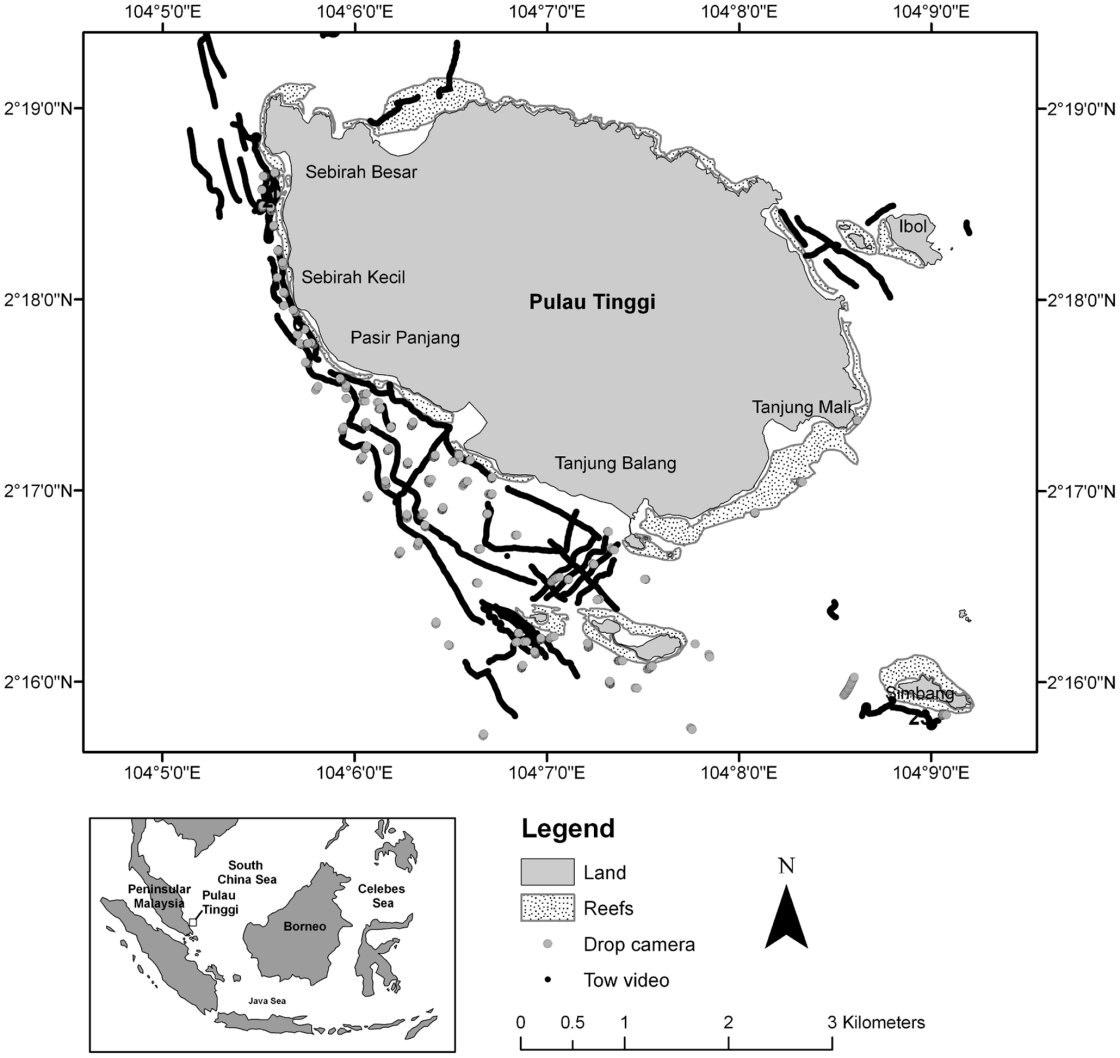
Location of tow video, drop video, and seagrass sampling points around Pulau Tinggi, southeast Peninsular Malaysia. Tow video points have converged to form tracks.

Towed video was used to characterize seagrass distribution along transects covering the range of sedimentary environments, water depth, and bathymetric features in the study area (Fig. 1). Using an underwater camera mounted on a glider frame and towed from a boat at a speed of 0.5–2.0 knots, visual recordings were made of the substrate. The boat position, heading, and boat speed were logged from a GPS and linked to the video frames at 2 second intervals along each transect. The camera was maintained at a height of <0.5 m from the seabed, with an approximate angle of 45 degrees from the substrate to enable species identification. The towed video footage was analysed by recording seagrass species composition at intervals of 2.5 m within the seagrass beds and 10 m outside seagrass beds. Identification was possible for all species except for *Halophila* spp. What is identified as *Halophila* spp. in this study comprises a complex predominantly of *H. ovalis* but also of *H. decipiens* and *H. minor.*

Drop video was deployed to provide additional data in areas between transects, thus reducing the clustering effect typically caused by a transect-sampling approach. The drop video system consisted of an underwater video in water-tight housing attached to a quadrat measuring 0.5×0.5 m. At each drop video point, the video camera was lowered onto the seabed randomly, ten times. This resulted in ten samples at each point, at an average distance of 1–2 meters apart. Seagrass species composition within the quadrat was recorded for each sample and added to the full data set comprising sampling points from both towed and drop video.

The main data set was divided into a training dataset (n = 8036) and a validation dataset (n = 2164). The former was used for variogram construction to obtain the variogram parameters (nugget, sill, range). The latter was used in cross validation to select the best model type and number of nested structures, based on (1) the predictive power of the models and on (2) the goodness-of-fit and parsimony of the models The final models presented here are those where the mean, median, standard deviation, mean squared and total errors of the predicted values are 0 or closest to 0 (best predictive power), and which have the lowest *AIC* (best goodness-of-fit and parsimony). All data are available on request from the first author.

### Indicator Semivariograms

The spatial structure of seagrass species was quantified by the indicator semivariogram [30] separately for each species to depict how the occurrence of a species varies as a function of distance. The indicator variogram is defined as the average squared semi-difference between pairs of observations [30]:
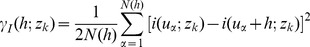
(1)


Where 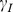 is the dissimilarity between data pairs separated by a distance of 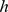; 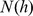 is the number of pairs of observations at distance 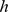; 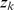 is the indicator threshold of species presence or absence at coordinates 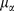. A low variance 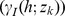 is closer to 0, corresponding to high correlation or continuity.

**Figure 2:**
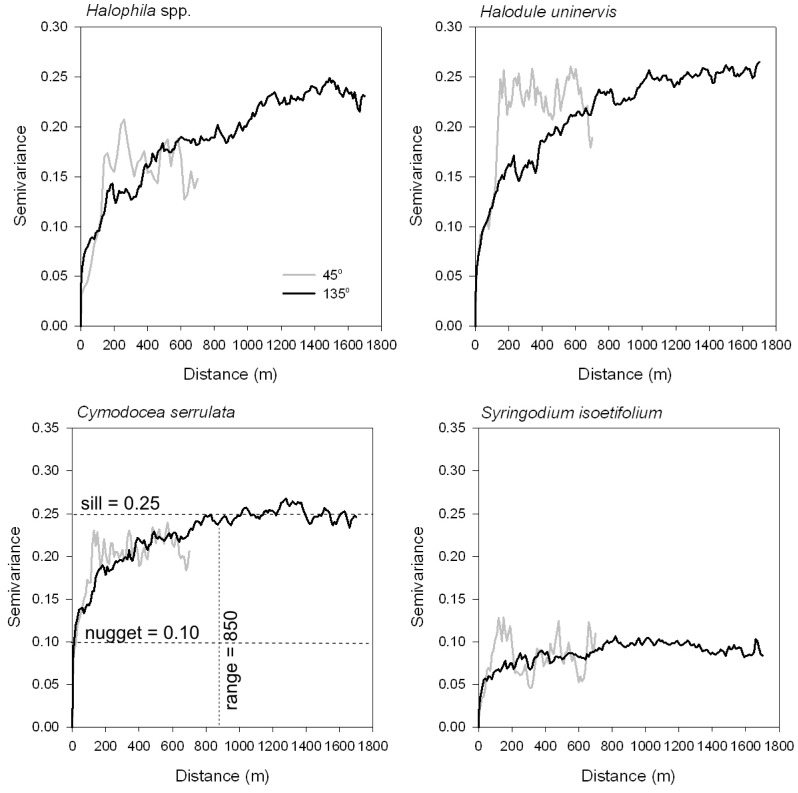
Directional variograms for presence/absence of four seagrass species in the along-shore (135°) and across-shore (45°) of Pulau Tinggi, Peninsular Malaysia. Other directions (0° = north-south and 90° = east-west) were modeled but are not shown. Variograms were plotted for half the maximum distance between points in each direction, lag 10 m, tolerance 5 m. The theoretical variogram structures (nugget, range and sill) are shown for *Cymodocea serrulata.*

Variograms were interpreted and fitted using one or a combination of the accepted models (spherical, exponential, Gaussian, nugget-effect) [31]. The variogram model parameters are (1) the *sill* (heterogeneity of species occurrence), i.e. the semivariance on the y-axis where the variogram reaches its asymptote, which is approximately the global variance of the data; (2) the *range* (maximum diameter of species patches or gaps), i.e. the distance on the x-axis where the semivariogram reaches its asymptote and beyond which all points are spatially uncorrelated; and (3) the *nugget*; i.e. the semivariance on the y-axis at the ordinate of the variogram due to measurement error and fine spatial-scale fluctuations in the property of interest [32] (Fig. 2). Geostatistical analyses were implemented in WinGslib 1.5.6.

Anisotropy is the property of having different patterns in different directions. To capture anisotropy, directional variograms were constructed for different azimuths, which were north-south (0°), northeast-southwest (45°), east-west (90°) and southeast-northwest (135°). Calculations were constrained to pairs of points oriented within 45 degrees of a particular azimuth (or 22.5° on either side of a point). Because variogram values are less reliable at large distances [33], variograms were computed up to half the maximum distance between sampling points. These distances were 1800 m (0°), 700 m (45°), 1300 m (90°) and 1700 m (135°).

Out of these four directional variograms, the two with the longest and shortest ranges were used to characterise spatial structure (question 1) and identify critical scales of study in the Linear Model of Coregionalization (question 2). These variograms were also used to produce kriged maps of species.

### Kriged Maps of Species

Ordinary indicator kriging was performed on the variogram model to provide a probability estimate of occurrence for each species in the study area. Indicator kriging predicts the odds of species presence in geographic space by using the parameters of the chosen variogram model to describe the spatial correlation structure with the following equation:
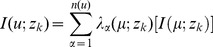
(2)


Where 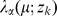 is the weight from the variogram modelling assigned to the indicator data 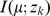 In this way, predictions of species occurrence can be made for unsampled locations based on occurrence data from samples in a local neighbourhood. If anisotropy is present in the dataset, kriging is performed using variograms of the axes of maximum and minimum continuity combined into a single model. Kriged maps were produced in grids measuring 5×5 m. Kriged output was exported to ArcGIS 9.3 for visualization and map production.

### Linear Model of Coregionalization (LMC)

The Linear Model of Coregionalization (LMC) was used to determine critical spatial scales in species distributions (question 2). The basic assumption of the LMC is that the variables of a model are generated by the superimposition of independent physical processes operating at different spatial scales [34]. In the case of co-occurring seagrass species, the separate species variograms may be fitted with variogram functions which allow inference of how strongly a corresponding scale-specific process influences the distribution of each species.

In this study, the LMC procedure was performed separately for the two directions of spatial continuity based on the assumption that these will each have different dominant spatial scales. The LMC procedure was applied by (1) computing individual species variograms, (2) choosing a suitable combination of basic variogram functions common to all species variograms, (3) fitting the selected model to the species variograms and (4) estimating the proportional contribution of the basic functions to each model, based on the rationale that comparison of the scale-specific sills reveals the proportion of variance accounted for at each scale [35]. The total variance at each spatial scale is formally expressed as [36]:
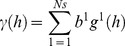
(3)


Where 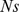 is the number of common variogram structures 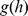, 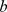 is the sill, and 1 indicates a particular spatial scale.

## Results

A total of 37 towed video transects amounting to 25 kilometres in length were collected. Out of the sampled 10,200 data points, 66% had seagrass (Table 1). *Halophila* spp. was the most frequently identified seagrass (65%), followed by *Halodule uninervis* (50%), *Cymodocea serrulata* (45%), and *Syringodium isoetifolium* (11%).

**Table 1:** Total number of frames obtained from tow and drop video, counts (*n*) and percentages (%) of frames with and without seagrass, and breakdown by species.

Video data	*n*	% of total frames
Total number of sampled frames	**10200**	
Tow video (∼2.5 m intervals)	9586	
Drop video (∼1.0 m intervals)	614	
Seagrass present	**6695**	**66**
Seagrass species		
*Halophila* spp.	6641	65
*Halodule uninervis*	5065	50
*Cymodocea serrulata*	4607	45
*Syringodium isoetifolium*	1096	11
Seagrass absent	**3505**	**34**

### Structure of Directional Variograms

All variograms showed strong spatial dependence with obvious sills (Fig. 2), indicating that these species were distributed in clearly defined patches or aggregations, but were structured differently in different directions. For all species, the axes of maximum and minimum continuity were 135° (along-shore direction) and 45° (across-shore direction). In the along-shore, range distances were shortest for *C. serrulata* and *S. isoetifolium,* while *Halophila* spp and *H. uninervis* extended over longer distances (Table 2). Because these species co-occur, the anisotropy ratios indicate that species distribution is spatially nested. *Halophila* spp. (*H. ovalis, H. decipiens* and *H. minor*) and *H. uninervis* were present in all patches from edge to edge, with the widest average patch size measuring around 1,000–1,400 m in the along-shore and 250–280 m in the across-shore. In patch interiors are *C. serrulata* and *S. isoetifolium* with average patch sizes of 800–850 m along-shore and 150–200 m across-shore.

**Table 2:** Parameters of models fitted independently to variograms of the four seagrass species in Pulau Tinggi, Malaysia.

Species	*C_o_*	*C_1_*	*C_2_*	*Sill*	*a_1_*	*a_2_*	NSR	AR
Along-shore (135°)								
*Halophila* spp	0.060	0.170*_Exp_*	–	0.230	1400	–	26	5.0
*H. uninervis*	0.060	0.190*_Exp_*	–	0.250	1000	–	24	4.0
*C. serrulata*	0.100	0.150*_Exp_*	–	0.250	850	–	40	4.3
*S. isoetifolium*	0.020	0.030*_Sph_*	0.045*_Exp_*	0.095	50	800	20	5.3
Across-shore (45°)								
*Halophila* spp	0.023	0.045*_Sph_*	0.092*_Sph_*	0.160	220	280	14	–
*H. uninervis*	0.040	0.030*_Sph_*	0.160*_Sph_*	0.230	30	250	17	–
*C. serrulata*	0.070	0.025*_Sph_*	0.115*_Sph_*	0.210	50	200	23	–
*S. isoetifolium*	0.015	0.030*_Sph_*	0.035*_Hole_*	0.080	130	150	19	–

All variograms required a nugget component plus an additional exponential, spherical or sine wave component. These are indicated as *Exp, Sph* and *Hole,* respectively.

*C_o_* = nugget, *C_1_* = variance contribution for the 1^st^ structure, *C_2_ = *variance contribution for the 2^nd^ structure, *a_1_* = range for the 1^st^ structure (m), *a_2_* = range for the 2^nd^ structure (m), NSR (nugget-to-sill ratio) = (*C_0_/Sill*)*100, AR = anisotropy ratio (*a_1_* in 135°: *a_2_* in 45°).

The directional variograms were well-structured in the along-shore direction, in that the semivariances increased gradually to reach a sill (Fig. 2). In the across-shore direction, variograms also reached their sills, but undulations were evident, indicating patchiness and repetition of patches in species distribution across nested spatial scales. All variograms required a nugget component plus an additional spherical or exponential component (Table 2), suggesting that taxa occurrence was influenced by at least 2–3 dominant processes.

**Figure 3:**
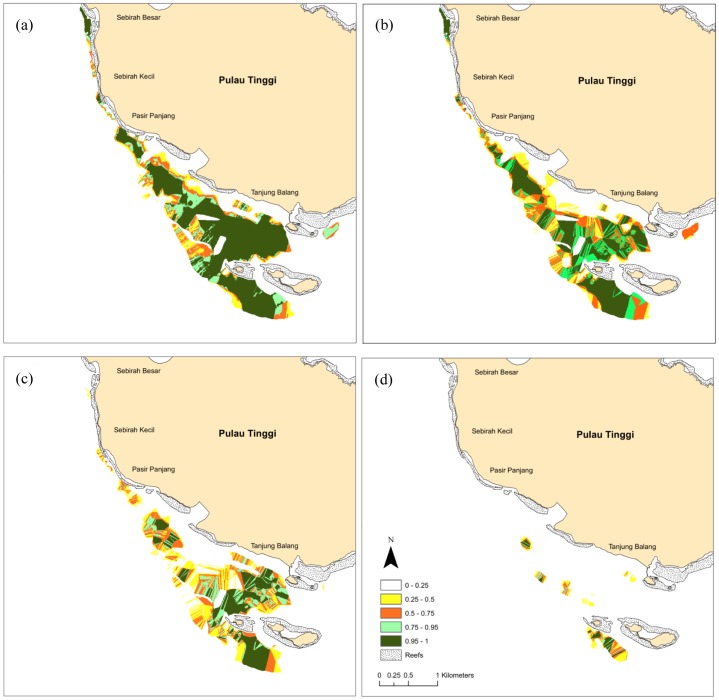
Probability estimates for the occurrence of (a) *Halophila* spp. (b) *Halodule uninervis* (c) *Cymodocea serrulata*, and (d) *Syringodium isoetifolium* in the southwest of Pulau Tinggi, Malaysia, produced by ordinary kriging in 5 m cells.

The kriged maps show a nested pattern of patch sizes and location, with *Halophila* spp. and *H. uninervis* having large extents, and *C. serrulata* and *S. isoetifolium* with smaller extents (Fig. 3). These species co-occur with the highest variability mostly at meadow edges, suggesting strong influence of environmental gradients on species distribution. Striped patterns in the kriged maps are an artefact of the sampling protocol, where strong currents made it difficult to orient transects in the across-shore direction.

### Spatial Structure of Species at Different Scales

**Figure 4:**
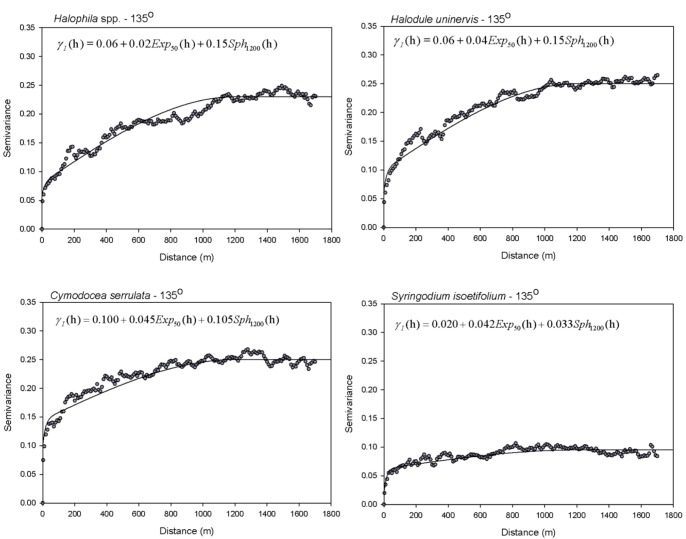
Sample variograms (dots) and the fitted Linear Model of Coregionalization (solid lines) of seagrass species presence/absence in the along-shore direction (135°) in Pulau Tinggi, Malaysia. Sample variograms are displayed with a lag of 10 m and a tolerance of 5 m.

The Linear Model of Coregionalization (LMC) was constructed for the four taxa as a group based on model parameters of the individual species variograms in Table 2. The main contributing spatial scales in the along-shore direction were <2.5 m (micro-scale), 2.5–50 m (fine-scale) and >50 m (broad-scale). A nugget component plus nested exponential and spherical components provided the best fit (Fig. 4, Table 3). Occurrence of *Halophila* spp and *H. uninervis* were dominated by broad-scale processes, with variance contributions between 60–65% (Table 4a). *C. serrulata* had equal contributions from micro-scale (40%) and broad-scale processes (42%). *S. isoetifolium* had the largest contributions from fine scale (42%) and broad scale (35%) processes.

**Table 3:** Parameters of the linear coregionalization for four seagrass species in Pulau Tinggi, Malaysia in the two principal directions of continuity.

Spatial scales	Model	Sill			
		*Halophila* spp.	*Halodule uninervis*	*Cymodocea serrulata*	*Syringodium isoetifolium*
*Along-shore (135*°*)*					
Micro-scale (<2.5 m)	Nug	0.060	0.060	0.100	0.020
Fine-scale (2.5–50 m)	Exp	0.020	0.040	0.045	0.042
Broad-scale (>50 m)	Sph	0.150	0.150	0.105	0.033
*Across-shore (45*°*)*					
Micro-scale (<2.5 m)	Nug	0.023	0.040	0.070	0.015
Fine-scale (2.5–140 m)	Sph	0.000	0.000	0.045	0.065
Broad-scale (>140 m)	Sph	0.137	0.190	0.095	0.000

Nug = nugget, Exp = exponential model, Sph = spherical model.

**Table 4:** Percent of total variance for each species explained at each spatial scale, fitted to the constraints of the Linear Model of Coregionalization in the (a) along-shore, and (b) across-shore.

Species	Micro-scale	Fine-scale	Broad-scale
(a) Along-shore direction	<2.5 m (%)	2.5–50 m (%)	>50 m (%)
*Halophila* spp.	26	9	65
*Halodule uninervis*	24	16	60
*Cymodocea serrulata*	40	18	42
*Syringodium isoetifolium*	21	42	35
(b) Across-shore direction	<2.5 m (%)	50–140 m (%)	>140 m (%)
*Halophila* spp.	14	0	86
*Halodule uninervis*	17	0	83
*Cymodocea serrulata*	33	21	45
*Syringodium isoetifolium*	19	81	0

**Figure 5:**
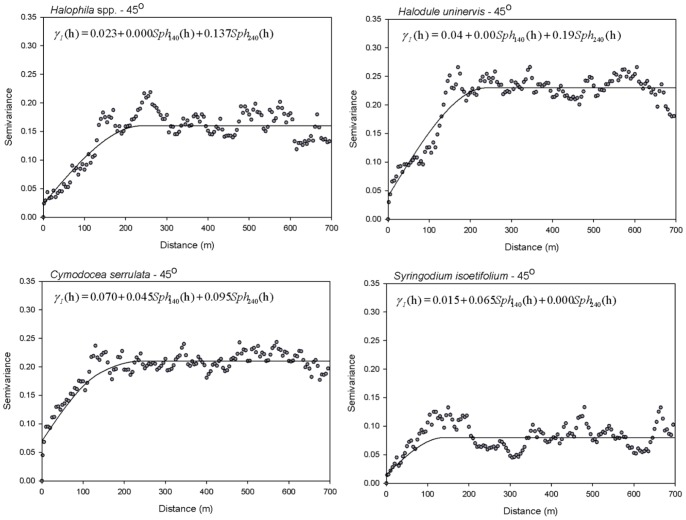
Sample variograms (dots) and the fitted Linear Model of Coregionalization (solid lines) of seagrass species presence/absence in the across-shore direction (45°) in Pulau Tinggi, Malaysia. Sample variograms are displayed with a lag of 5 m and a tolerance of 2.5 m.

In the across-shore direction, the main contributing spatial scales were <2.5 m (micro-scale), 2.5 m –140 m (fine-scale) and >140 m (broad-scale). The variograms were best fitted by a nugget component and a double spherical component (Fig. 5, Table 3). *Halophila* spp and *H. uninervis* had large variance contributions of 83–86% by broad-scale processes (Table 4b). The broad-scale had a large influence (45%) on *C. serrulata,* as did the micro-scale (33%). *S. isoetifolium* was dominated by processes at the fine-scale (81%), whereas the micro-scale and broad-scale had little to no influence on its occurrence.

## Discussion

### 1. Spatial Patterns were Directional, Nested and had Multiple Structures

In the forereefs of Pulau Tinggi, seagrass species had spatial patterns that were directional, nested, and consisted of multiple structures. The largest difference in directionality was between the along-shore and across-shore, pointing to the presence of gradients oriented predominantly along those axes. Species distributions were characterized as “nested” because *C. serrulata* and *S. isoetifolium* were present mainly in meadow interiors while *Halophila* spp and *H. uninervis* occupied the larger portion of the gradient. Nestedness implies spatial organization in response to a common environmental gradient interacting with biological mechanisms such as differential colonization abilities and physiological limits of species. Also, the variograms of each of the species were fitted best with more than one model structure, suggesting that species were influenced by multiple additive processes acting across multiple scales.

### 2. Critical Spatial Scales are Linked to Species Size

By quantifying the spatial structure of the co-existing species, we detected critical scales for each of them. The LMC showed each species to likely be influenced by 2–3 processes across a range of scales. The dominant nested spatial scales in the along-shore were <2.5 m (micro-scale), 2.5–50 m (fine-scale), and >50 m (broad-scale); and in the across-shore were <2.5 m (micro-scale), 2.5–140 m (fine-scale), and >140 m (broad-scale).

**Figure 6:**
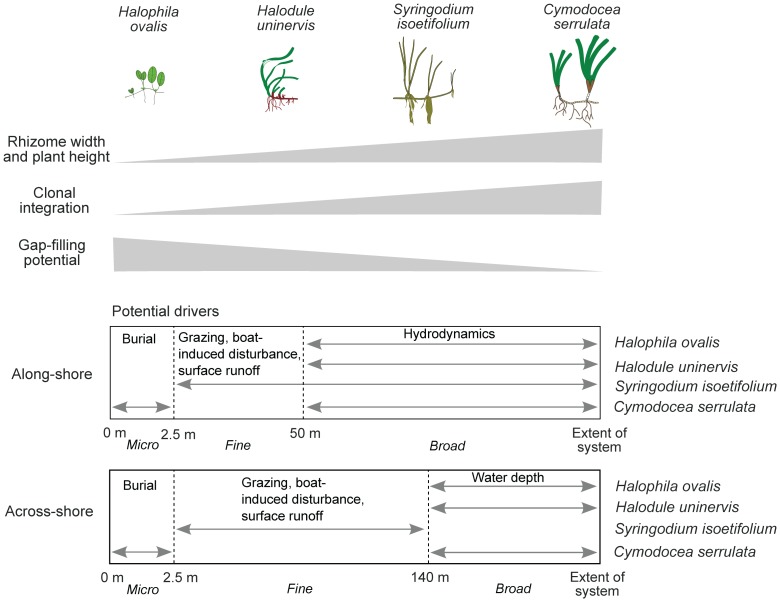
Relative characteristics of the four seagrass species in Pulau Tinggi, Malaysia, including rhizome width and plant height (species size), degree of clonal integration and gap-filling potential. Potential drivers in the along-shore and across-shore are shown. Arrows denote the distances at which a species is most strongly influenced (>30% variance in the Linear Model of Coregionalization).

Size-based rules were evident. In ascending order of species size and descending order of structure based on the spatial scale of environmental processes and drivers are *H. ovalis, H. uninervis, S. isoetifolium* and *C. serrulata* [37]. *Halophila* spp and *H. uninervis* were likely to be influenced predominantly by broad-scale processes; *S. isoetifolium* by fine-scale processes; and *C. serrulata* equally by micro and broad-scale processes (Table 4). Thus, this spatially-explicit approach suggests that large and localised species were influenced by finer-scale processes across a wider range of spatial scales, whereas small, widespread species appeared to be driven almost entirely by broad-scale processes (Fig. 6).

Other studies have also implied this pattern. In a study linking species diversity and shoot density to a siltation gradient at the broad spatial scale of hundreds of kilometres, *H. ovalis* was found to be the least tolerant of variation in siltation, whereas *S. isoetifolium* and *C. serrulata* had weaker responses [38]. In contrast, interspecific competition at the scale of centimetres to metres did not result in significant variation in *H. ovalis,* whereas it did for larger species such as *Thalassia hemprichii* and *S. isoetifolium* [8], [39]. The idea here is that there is a size-dependent relationship between species and the spatial scale of their environmental drivers. Thus, intrinsic plant traits, which in seagrasses are size-dependent [37], [40], interact with external drivers to produce structure in the spatial distribution of species.

### 3. Potential Scale-specific Drivers

With critical spatial scales identified by the LMC, we propose hypotheses about the dominant drivers on species distribution in the study area, on the basis of matching spatial scales.

#### Micro-scale (<2.5 m)

Variation in species occurrence at <2.5 m could be the result of biotic disturbances that cause a reorganisation of seagrass species and the placement of their shoots. These include grazing by dugongs and turtles and bioturbation by infauna such as crustaceans, echinoderms, molluscs, oligochaetes and polychaetes, all of which are common and abundant in this region [41]. Wherever they graze, dugongs impact seagrasses by removing whole plants and causing seagrasses to change their growth patterns and morphology [42], and by selecting for fast-growing species such as *H. ovalis* [43]. Similarly, infauna have the capacity to cause localised substrate disturbance that could have large cumulative effects on the structure of marine ecosystems [44], including seagrass.

However, burrowing activity by infaunal shrimp was the most appreciable biotic driver in our site, evident from the widespread presence of shrimp sediment mounds. These mounds occurred at densities of 1–2 m^−2^ in Pulau Tinggi, and elsewhere in Southeast Asia have been reported in densities of 2–3 m^−2^ on backreef seagrass meadows [45], [46]. Sediment mounds result not only in the burial of seagrasses, but possibly also in the germination of seeds exposed by the process of bioturbation [21]. Thus, when coupled with species-specific responses of seagrasses to burial, shrimp mounds may account for much of the random, unpredictable patterns of seagrass distribution observed as a nugget effect in this study.

The variance contribution to *C. serrulata* was double that of the other species at the micro-scale suggesting greater variability in its occurrence as a result of micro-scale processes such as sediment burial. However, past experimental evidence suggested quite the opposite – that *C. serrulata* should be the least variable of all species at the micro-scale because it is the one most likely to survive burial and to remain present in the landscape [37]. Its internal growth program may provide an explanation: as the largest species in this study, *C. serrulata* elongates less rapidly, has larger spacing between successive shoots, and branches less frequently and at more shallow angles than the others [4], [47]. Thus, *C. serrulata* may be good at spreading across substrate but does not fill up gaps caused by sediment mounds as efficiently and quickly as *H. ovalis, H. uninervis* and *S. isoetifolium*. This has also been demonstrated in a cross-species removal experiment [6]. Such a growth program could result in the random occurrence of *C. serrulata* at the micro-scale despite its ability to out-survive the others when buried under sediment.

#### Fine-scale (2.5–50 m along-shore, 2.5–140 m across-shore)

Fine-scale drivers were considerably more difficult to identify because of the wide range in distances. However, the type of variogram models used is informative about patch characteristics. The exponential model in the along-shore (Table 3) indicates that patches have an irregular extent usually attributed to stochastic processes [48]. Irregular gap formation at this scale could have been caused by the grazing of mega fauna, sediment erosion and deposition both natural and caused by boat movement and anchoring, and surface runoff outlets from land which cause changes in salinity and nutrient input. In the across-shore direction, the spherical model provided the best fit. Thus, drivers in this direction resulted in species patches that were fairly regular.

#### Broad-scale (>50 m along-shore, >140 m across-shore)

Variation in species occurrence at the broad-scale is potentially driven by hydrodynamics in the along-shore. Pulau Tinggi has diurnal tides, with a daily range of 0.5 to 1.5 m (Malaysian Meteorological Department, 2010). During the twice-daily change of tides, the flow of water current was in the along-shore direction at velocities of more than 50 cm s^−1^. In Pulau Tinggi, hydrodynamics may increase plant dispersal between beds and cause substrate disturbance, resulting in between-meadow differences in seagrass populations, especially for *Halophila* spp. and *H. uninervis*.

In the across-shore direction, the depth gradient (light gradient) is a possible broad-scale driver. At the landward meadow edge where water depth is around 3 m, photosynthetically active radiation (PAR) averages 37% of surface irradiance. The meadow slopes down gradually to around 10 m depth where PAR averages 15% of surface irradiance, beyond which no seagrasses occur [29]. Because *Halophila* spp and *H. uninervis* possess small rhizomes, they have small respiratory demands [49] and they do not integrate resources as well as larger species such as *S. isoetifolium* and *C. serrulata* [40], [50]. These are size-specific traits which result in small species having greater sensitivity to environmental changes that occur over the broad spatial scale, such as a reduction in light along a gradient of water depth. Light and seasonal environmental variability have previously been shown to affect resource availability for small species [51].

The conceptual model proposed for seagrass species distribution in Pulau Tinggi is based on their spatial structure (Fig. 6). However, it would be simplistic to assume close associations between pattern and process on the basis of matching scales and variogram structure alone, i.e. different ecological processes may present similar variogram structures or one single ecological process may present many different structures. Therefore, these models are meant merely to provide a framework within which competing hypotheses about underlying drivers may be constructed and further tested.

## Conclusions

The forereef seagrass system in Pulau Tinggi exhibits clear spatial structure, in that patterns of species distribution were directional, nested and had multiple structures. We found evidence for size-based rules in the distribution of marine plant communities in response to their underlying drivers, where small, widespread species such as *Halophila* spp and *H. uninervis* were most influenced by broad-scale processes whereas large, localised species such as *S. isoetifolium* and *C. serrulata* were most influenced by finer-scale processes over a wider range of distances.

This study demonstrates the potential of using spatial structure to provide further clues about the underlying drivers of species distribution. Based on directionality and the scales of influence, we proposed a range of potential drivers of seagrass structure in the study area. Hydrodynamics and water depth are potentially the broad-scale drivers that operate at distances of >50 m in the along-shore and >140 m in the across-shore, providing support for the view that these are the prime drivers of meadow extent [1]. Sediment burial under bioturbation mounds is a likely driver at distances of <2.5 m. Gap formation caused by grazing, disturbance from boating activities and surface runoff potentially influences the spatial structure of seagrass species at distances of 2.5–50 m in the along-shore and 2.5–140 m in the across-shore.
